# *Ralstonia mannitolilytica* sepsis: a case report

**DOI:** 10.1186/s13256-019-2235-0

**Published:** 2019-10-26

**Authors:** Michael Owusu, Godfred Acheampong, Augustina Annan, Kwadwo Sarfo Marfo, Isaac Osei, John Amuasi, Nimako Sarpong, Justin Im, Ondari D. Mogeni, Hsin-Ying Chiang, Chih-Horng Kuo, Hyon Jin Jeon, Ursula Panzner, Se Eun Park, Florian Marks, Ellis Owusu-Dabo, Yaw Adu-Sarkodie

**Affiliations:** 1 Department of Medical Diagnostics, Kwame Nkrumah University of Science and Technology, Kumasi, Ghana; 2 Kumasi Centre for Collaborative Research in Tropical Medicine, Kwame Nkrumah University of Science and Technology, Kumasi, Ghana; 3 Department of Theoretical and Applied Biology, Kwame Nkrumah University of Science and Technology, Kumasi, Ghana; 4 Department of Global Health, School of Public Health, Kwame Nkrumah University of Science and Technology, Kumasi, Ghana; 5 Agogo Presbyterian Hospital, Agogo, Ashanti Region Ghana; 6 Department of Epidemiology, International Vaccine Institute, Seoul, Republic of Korea; 7 Institute of Plant and Microbial Biology, Academia Sinica, Taipei, Taiwan; 8 Department of Clinical Microbiology, Kwame Nkrumah University of Science and Technology, Kumasi, Ghana

**Keywords:** *Ralstonia mannitolilytica*, Sepsis, Nonfermenting gram-negative rods, Case report, 16S rRNA

## Abstract

**Background:**

*Ralstonia mannitolilytica* is an emerging opportunistic pathogen that is associated with severe disease, including septic shock, meningitis, and renal transplant infections. Reports on this pathogen are limited, however, especially on the African continent.

**Case presentation:**

A 2-year-old Akan child was presented to a hospital in the northeastern part of Ghana with a 1-week history of fever and chills. We identified *Ralstonia mannitolilytica* in her blood culture using both conventional and 16S ribosomal deoxyribonucleic acid (rDNA) techniques. The patient’s condition improved clinically upon treatment with cefuroxime.

**Conclusion:**

Our report highlights the potential of *Ralstonia mannitolilytica* to cause sepsis and thus emphasizes the need for improved laboratory diagnosis and evidence for use of appropriate antibiotics in rural settings of Africa, where presumptive treatment using antimicrobial agents is rife.

## Background

Nonfermenting gram-negative rods are one of the commonest causes of nosocomial infections in clinical environments. The major opportunistic pathogens in this group are *Acinetobacter baumanii*; *Stenotrophomonas maltophilia*; and other oxidase-positive bacteria, such as *Pseudomonas aeruginosa* and *Burkholderia cepacia*.

*Ralstonia mannitolilytica* (*R. mannitolilytica*) is another emerging opportunistic pathogen within the nonfermenting gram-negative bacillus group that is present in both hospital and environmental settings [1]. *R. mannitolilytica* had previously been referred to as “*Pseudomonas thomasii*” and *R. pickettii* biovar 3/“thomasii” [2]. Although clinical infections with this pathogen are rare, disease progression to severity tends to be more serious once individuals are exposed. A large oncology hospital in Rome recently reported *R. mannitolilytica* infections among 12 oncology outpatients attending a day ward [3]. China similarly reported three cases of bloodstream infections with *R. mannitolilytica* [4], and Belgium recorded two clinical cases of recurrent meningitis on an implanted intraventricular catheter and an infected hemoperitoneum [5].

Although these infections could potentially occur in Africa, especially as a result of poor environmental conditions and infection prevention control practices, reports on them are rare, perhaps because of limited diagnostic capacity. We report a case of *R. mannitolilytica* sepsis in a 2-year-old child at a rural hospital in the Ashanti Region in Ghana.

## Case presentation

A 2-year-old Akan child was presented to a rural hospital in the Ashanti Region of Ghana with a 1-week history of fever that had been controlled with the use of acetaminophen syrup. On examination, the patient weighed 19.0 kg, was anicteric, and looked pale. Her heart rate was 132 beats per minute with normal heart sounds. Results of her respiratory and abdominal examinations were also normal. On the basis of clinical findings, provisional diagnoses of malaria and sepsis were made. Pending laboratory results, she was empirically treated with 650 mg of intravenous cefuroxime three times daily and 50 mg of intravenous artesunate at 4-hourly intervals.

Her full blood count investigation showed a hemoglobin concentration of 9.2 g/dl, total white blood cell count of 5.6 × 10^3^ cells/μl, and platelet count of 81 × 10^9^ cells/μl. The result of her malaria smear test was positive.

**Fig. 1:**
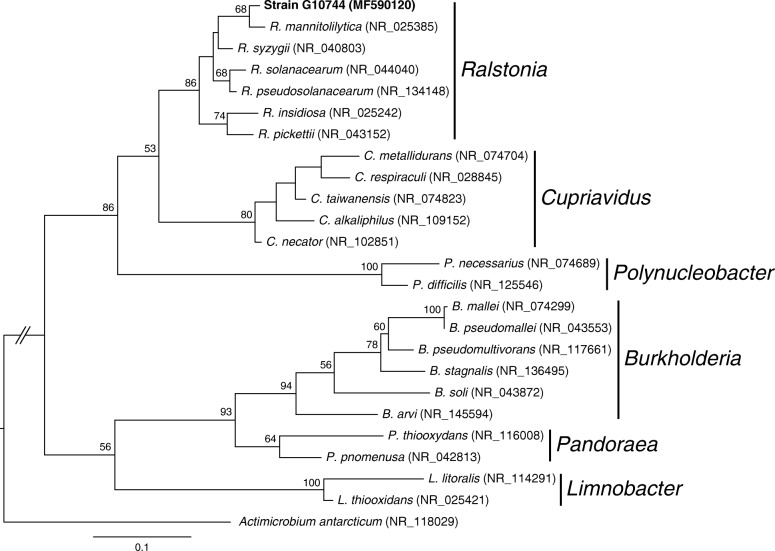
Maximum likelihood phylogeny based on 16S rRNA gene sequences. The GenBank accession number for each sequence is provided in parentheses next to the species name abbreviation The genus name is listed to the right of all applicable entries. The levels of bootstrap support based on 1000 replications are labeled above internal branches; only values greater than 50% are listed. On the basis of a BLASTN sequence similarity search against the NCBI 16S rRNA sequence database, the strain G10744 is most similar to the type strain of *Ralstonia mannitolilytica* (1350/1360 = 99.3% sequence identity). Other representative species from the family Burkholderiaceae are included to infer the phylogenetic placement of G10744. The sequence from *Actimicrobium antarcticum* (family Oxalobacteraceae) is included as the outgroup to root the phylogeny

Urine and stool culture results were negative for any bacteria. The blood culture, however, yielded a nonfermenting gram-negative bacterium. The bacterium was processed for identification using the analytic profile index (API) (bioMérieux, Marcy-l’Étoile, France) specific to non-Enterobacteriaceae (API-20NE). This showed an Identification number (ID) of 0045477, which was consistent with *R. pickettii.* Further confirmation was achieved using the 16S ribosomal ribonucleic acid (rRNA) method described in our previous study [6]. In summary, deoxyribonucleic acid (DNA) was extracted from pure culture of the bacterium using the SpheroLyse extraction kit (Hain Lifesciense GmbH, Nehren, Germany). The 16S rDNA was amplified using primer pair 8F and 1492R, and the resulting sequence was checked using DECIPHER (version 2.2.0). On the basis of the BLASTN sequence similarity search against the NCBI 16S rRNA sequence database, the strain was found to be the strain type of *R. mannitolilytica* (1350/1360 = 99.3% sequence identity) (Fig. 1). The sequence has been deposited in the National Center for Biotechnology Information database [GenBank:MF590120].

Antimicrobial susceptibility testing of the isolate showed resistance to ampicillin and sensitivity to gentamicin, cefuroxime, ciprofloxacin, ceftriaxone, and cotrimoxazole. The patient’s condition improved clinically upon treatment with cefuroxime.

## Discussion and conclusion

*R. mannitolilytica* infections are not common in clinical settings. The few reported cases have been of the sister genus, *R. pickettii*. Although less frequent, the few documented cases are severe, with reported incidences in hospital outbreaks, bacteremia and bacteriuria, meningitis, renal transplant infection, and hemoperitoneum infection [4, 5, 7]. In rare cases, *R. mannitolilytica* has been isolated from contaminated oxygen delivery devices [1]. Our patient was a 2-year-old child residing in a rural part of Ghana. She had no known history of any underlying chronic disease or immunological suppression apart from her presentation of fever and chills. The presence of this bacterium in the blood is of importance, being the first such report from West Africa. Although it might be difficult to determine the extent of disease severity on account of malaria or sepsis, the isolation of a pure strain of the bacterium in blood suggests a pathogenic association with sepsis. Our report is not different from a recent review of three infants from China who similarly presented with chills and fever [4]. Their cases progressed to septic shock, however, with symptoms including increased heart rate and decreased urine output. We also observed reduced sensitivity in the accuracy of speciation by the API. Although the API identified this bacterium as *R. pickettii*, the 16S rDNA technique revealed this as *R. mannitolilytica.* Other authors have similarly reported variations and inconsistencies in the use of standard biochemistry-based techniques for identification of *Ralstonia* because these techniques share similar biochemical patterns [8]. This emphasizes the importance of using molecular techniques as a supporting diagnostic confirmation, especially for nonfermenting bacteria.

Antimicrobial susceptibility testing also showed susceptibility to antibiotics, including gentamicin, cefuroxime, ciprofloxacin, ceftriaxone, and cotrimoxazole. Treatment and management of *Ralstonia* spp. has been reported as challenging because of their intrinsic resistance to inducible β-lactamases [9]. The isolated bacterium did not exhibit this form of resistance. A more structured epidemiological study would be helpful to further evaluate this occurrence.

This report shows that *R. mannitolilytica* might be more widely distributed than previously thought. Active surveillance is therefore recommended to further understand its epidemiology, public health impact, and geographic distribution.

## Data Availability

All data generated or analyzed during this study are included in this published article, and the sequences have been deposited in the GenBank database.

## Abbreviation

API: Analytic profile index
